# Structural and Optical Properties of Silicon Carbide Powders Synthesized from Organosilane Using High-Temperature High-Pressure Method

**DOI:** 10.3390/nano11113111

**Published:** 2021-11-18

**Authors:** Evgeny A. Ekimov, Vladimir S. Krivobok, Mikhail V. Kondrin, Dmitry A. Litvinov, Ludmila N. Grigoreva, Aleksandra V. Koroleva, Darya A. Zazymkina, Roman A. Khmelnitskii, Denis F. Aminev, Sergey N. Nikolaev

**Affiliations:** 1 Institute for High Pressure Physics, Russian Academy of Sciences, Kaluzhskoe Shosse, 14, Troitsk, 108840 Moscow, Russia; ekimov@hppi.troitsk.ru (E.A.E.); mkondrin@hppi.troitsk.ru (M.V.K.); 2 P.N. Lebedev Physical Institute, Russian Academy of Sciences, Leninsky Prospect, 53, 119991 Moscow, Russia; litvinovd@lebedev.ru (D.A.L.); ln.grigorjeva@physics.msu.ru (L.N.G.); zazymkina_darya@mail.ru (D.A.Z.); roma@lebedev.ru (R.A.K.); amdenis@yandex.ru (D.F.A.); nikolaev-s@yandex.ru (S.N.N.); 3 Faculty of Physics, Lomonosov Moscow State University, Leninskiye Gory 1, 119991 Moscow, Russia; koroleva.phys@mail.ru

**Keywords:** crystal morphology, etching, growth models, nanostructures, X-ray diffraction, semiconducting silicon compounds

## Abstract

The development of new strategies for the mass synthesis of SiC nanocrystals with high structure perfection and narrow particle size distribution remains in demand for high-tech applications. In this work, the size-controllable synthesis of the SiC 3C polytype, free of sp^2^ carbon, with high structure quality nanocrystals, was realized for the first time by the pyrolysis of organosilane C_12_H_36_Si_6_ at 8 GPa and temperatures up to 2000 °C. It is shown that the average particle size can be monotonically changed from ~2 nm to ~500 nm by increasing the synthesis temperature from 800 °C to 1400 °C. At higher temperatures, further enlargement of the crystals is impeded, which is consistent with the recrystallization mechanism driven by a decrease in the surface energy of the particles. The optical properties investigated by IR transmission spectroscopy, Raman scattering, and low-temperature photoluminescence provided information about the concentration and distribution of carriers in nanoparticles, as well as the dominant type of internal point defects. It is shown that changing the growth modes in combination with heat treatment enables control over not only the average crystal size, but also the LO phonon—plasmon coupled modes in the crystals, which is of interest for applications related to IR photonics.

## 1. Introduction

Silicon carbide (SiC) is the most widely used non-oxide ceramic, which has applications in many industrial fields, due to its special semiconducting and spin-related properties, high mechanical strength and hardness, high thermal conductivity, resistance to corrosion and thermal shock, etc. [1,2,3,4,5,6,7,8,9,10,11,12]. In modern literature, various SiC-based nanomaterials are also actively discussed in terms of their usage in manufacturing implantable microelectrodes, highly porous membranes [8], biosensors [8,9,10,11,12], micro- (MEMS) and nanoelectromechanical systems (NEMS) [5], and heat-resistant coatings [11]. A number of methods to synthesize nano- and micropowders have already been developed, including growth from hydrogen silicone oil [6], mechanical grinding [11,12], fast carbothermal synthesis [13,14,15], combustion synthesis [16], microwave synthesis [17], pyrolysis of polymers [18], sol-gel processes [19], CVD [20], and laser synthesis [21]. All of these processes have their own advantages and disadvantages, associated with the cost of precursors, synthesis conditions, the purity degree of the materials obtained, and others [7]. The problem of developing processes for the synthesis of nanosized SiC, which has a high crystallinity degree, uniform particle size distribution, and is free of inclusions and structural defects, is still urgent [22]. In contrast to low-pressure synthesis paths [23,24], HPHT synthesis in the diamond stability region can ensure the formation of SiC nanopowders that are free of sp^2^ carbon. It is known that HPHT conditions are favorable for producing the SiC 3C polytype in the Si-C system [25,26], although its formation stability under pressure in the C-H-Si growth medium has not been investigated yet.

Special attention is paid to "core–shell" composite spherical nanostructures, which consist of a SiC core (inner material) and a shell (outer layer material). The latter can also consist of SiC, but it can have different properties and show different behavior. Of particular interest is the situation in which either the core or the shell is characterized by a high free carrier concentration, which enables such particles to be considered as plasmon resonators for the mid-IR range [27]. It should also be noted that in polar SiC nanocrystals, localized phonon polaritons can exist along with localized surface plasmons [28,29]. In undoped nanocrystals, the excitation frequencies occupy the frequency range between the transverse and longitudinal lattice resonances [25]. The near fields of such excitations can also be of interest for the needs of IR photonics [30,31,32].

In this work, it is shown that synthesis from organosilanes at high pressures (HTHP) is one of the possible methods for obtaining nanopowders of 3C silicon carbide that are free of sp^2^ carbon, with high crystal quality and relatively low size dispersion. In this case, the synthesis temperature is the parameter that controls the particle size. The developed method also enables particles with well-defined phonon–plasmon resonance to be obtained, the structure of which can be tuned by heat treatment. This paper discusses the structural features of the synthesized powders, the composition of their background impurities, and the optical properties, with an emphasis on particles with pronounced phonon–plasmon resonance.

## 2. Materials and Methods

**Figure 1:**
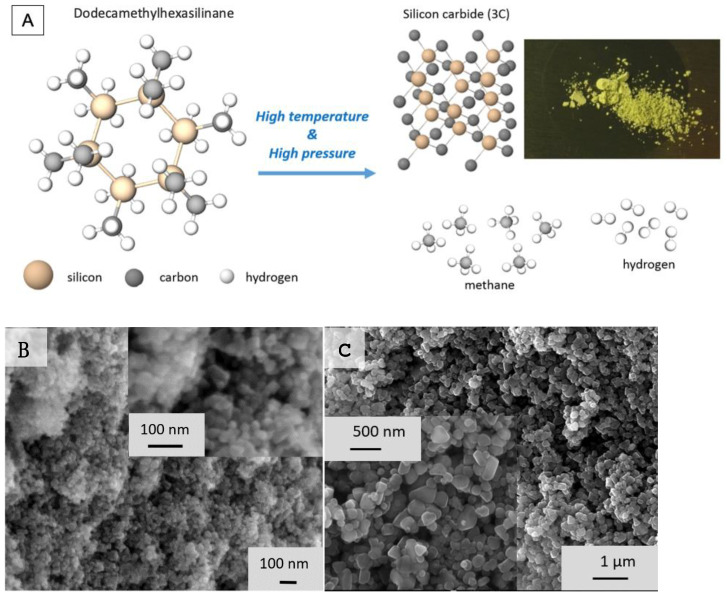
(**A**) The structure of dodecamethylhexasilinane (C_12_H_36_Si_6_) molecule and scheme of SiC formation in HTHP reaction. Photos in the right part of (**A**) show an example of 3C SiC powders obtained at 2000 °C. (**B**,**C**) SEM images of 3C SiC powders synthesized at temperatures of 1250 °C and 1400 °C, respectively.

In this work nanosized and submicron SiC crystals were obtained by pyrolysis of an individual compound dodecamethylhexasilinane C_12_H_36_Si_6_ (organosilane), Sigma-Aldrich Chemie GmbH, Taufkirchen, Germany, at high pressures and temperatures (Figure 1A). The choice of this compound for HPHT synthesis was due to its high purity (99.9% metal basis) and favorable chemical composition. On the one hand, dodecamethylhexasilinane contains excess carbon for silicon carbide formation, which can ensure the absence of unreacted silicon in the synthesis products. On the other hand, hydrogen is in excess in relation to carbon for methane formation, which should lead to the absence of free carbon in the growth medium. We used a toroid-type high-pressure cell for the synthesis at pressures of 8–9 GPa and temperatures up to 2000 K. The heating elements of the cell consisted of graphite (graphite, MGOSCH 99.9999%, NIIGrafit, Moscow, Russia) current leads and a titanium (99.99%, Sigma-Aldrich Chemie GmbH, Taufkirchen, Germany) crucible heater (capsule), which were in contact with a chemically inert container made of refractory zirconium oxide (99.9%, Sigma-Aldrich Chemie GmbH, Taufkirchen, Germany) [33]. The characteristic holding time at constant P and T was about 60 seconds. A uniaxial compression apparatus (homemade HPPI, Troitsk, Russia) and a high-pressure chamber of the “Toroid-15” type (homemade HPPI, Troitsk, Russia) created high pressures and temperatures. Dodecamethylhexasilinane tablets (2.5–3 mm thick and 6 mm in diameter) were pressed and loaded into capsules in air. The graphite current leads were separated from the surface of the hard-alloy (WC + Co) parts (homemade HPPI, Troitsk, Russia) of the high-pressure chamber by molybdenum (99.9% Sigma-Aldrich Chemie GmbH, Taufkirchen Germany) disks 0.2 mm thick to prevent their interaction. The temperature in the experiment was determined by a chromel–alumel thermocouple (homemade HPPI, Troitsk, Russia) up to 1350 °C. For higher synthesis temperatures, the temperature was estimated using a calibration dependence of the temperature in the reaction volume on the power of the electric current supplied for heating. The calibration dependence was previously found using a WRe 5/20 thermocouple (Contact of two wires: Wolfram 80%-Renium 20% wire and Wolfram 95%-Renium 5% wire). The titanium capsule started to interact with the contents of the cell, as well as the material of the container at temperatures exceeding 1300–1400 °C. For this reason, a graphite capsule was also used for the synthesis in the temperature range 1700–2000 °C. The mass of a sample obtained at temperatures above 800 °C was about 30–35 mg.

The determination of the elemental sample composition, as well as the morphology study of the synthesis products, was carried out using a JEOL JSM-6390LV, JEOL Ltd. Tokyo, Japan, electron microscope equipped with EDX microanalysis. Except for oxygen, impurities were not detected by EDX analysis; their concentration was less than the detection limit of 0.1–0.05 wt. % by the EDX method (see Figure S1 of Supplementary Materials). Quantitative atomic emission spectral analysis (ISP 30, ASMA-Pribor, Moscow, Russia) with use of standards shows presence of Ca (<10^−3^ mass%), Al (<10^−3^ mass%), Fe (<10^−3^ mass%), Mg (<10^−3^ mass%) and B (<10^−3^ mass%) in initial compound and samples obtained. X-ray diffraction studies (Cu K_α1_ radiation) were performed by using an imaging plate Guinier camera G670 (Huber), Huber Diffraktionstechnik GmbH, Rimsting, Germany.

## 3. Results and Discussion

### 3.1. Phase Transformations in C-H-Si System

**Figure 2:**
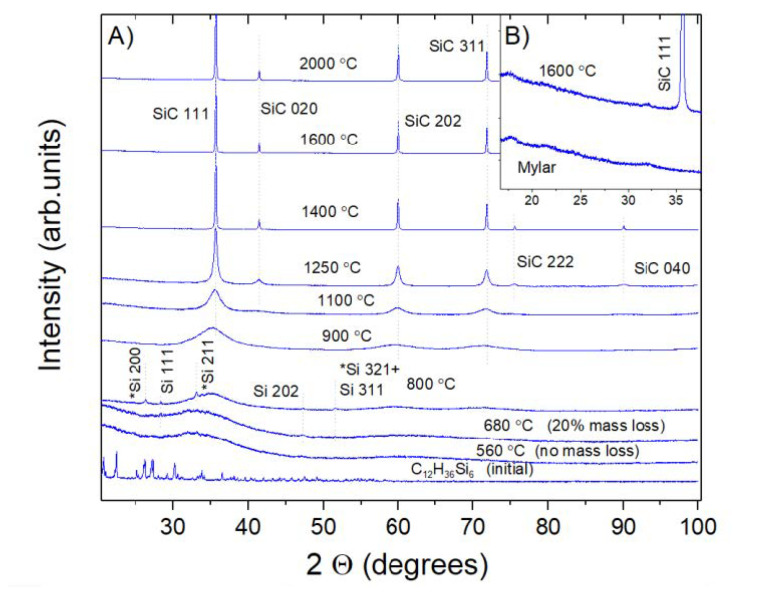
(**A**) Diffraction patterns of SiC powders synthesized at different temperatures by pyrolysis of C_12_H_36_Si_6_. (**B**) the XRD pattern background of one sample and Mylar sample holder with no signs of the strongest 002 graphite peak in the patterns.

The X-ray diffraction patterns of the synthesized samples are shown in Figure 2. Their analysis shows that the compound transforms into an amorphous matter at a temperature of 560 °C, without mass loss. Decomposition of the material begins at a temperature of about 680 °C, accompanied by sample mass loss of 20% and the formation of free silicon in the amorphous matrix with a changed composition. The formation of nanocrystalline silicon carbide, about 1.6 nm in size, is observed at 800 °C, while the sample contains an insignificant amount of free silicon in two cubic modifications (ordinary and Kasper’s phase). The average crystallite size of SiC increases intensively to about 150 nm with the rise in the synthesis temperature up to 1400 °C; however, at higher temperatures, 1600–2000 °C, further growth of the crystals becomes ineffective. The SiC crystallite sizes were estimated by the FWHM of 111 reflections, using the Scherrer formula, and are presented in Table 1, along with the lattice parameters calculated by the Rietveld method, with the use of NIST640c silicon standard. The absence of the strongest 002 line of graphite at 26 degrees in the diffraction patterns of the samples (Figure 2) implies that graphite does not form during the course of the synthesis. The calculated crystallite sizes are in good agreement with the sizes observed in electronic photographs (SEM) (see examples of sample morphology in Figure 1C and Table 1). An example of a crystal size distribution statistical analysis is given in Figures S2 and S3 of Supplementary Materials. Note that because of the small XRD line broadening, the error in determining its value becomes significant if the crystallite size is larger than 150 nm. In accordance with the SEM investigation, the sample obtained by synthesis at 1100 °C is characterized by agglomeration of the primary particles into 100–200 nm aggregates. At synthesis temperatures of 1250 °C, distinct ~20–30 nm crystallites are formed (see Figure 1C). At temperatures above 1350–1400 °C, 100–500 nm individual particles are formed, without noticeable size changes at further temperature increases up to 2000 °C.

The results related to the temperature-induced crystal growth of SiC can be interpreted in terms of recrystallization, driven by a decrease in the surface energy of the nanoparticles [34]. After the complete decomposition of the organosilane compound at about 800 °C, there is no longer a source of Si and C feeding crystal growth, except for the nanocrystals themselves.

Similarly to the growth of diamonds under pressure in the C-H growth system [35], SiC nanocrystals can grow due to the dissolution–precipitation of carbon and silicon in the C-H-Si growth medium, formed during the decomposition of dodecamethylhexasilinane, or due to the coalescence of the nanoparticles [34]. Our experiments show that with a particle size of more than 200–500 nm, a decrease in the surface-to-volume ratio no longer plays a key role as a driving force in the growth of SiC crystals, and crystal growth slows down. It is interesting to note that in experiments based on sintering SiC nanopowders under pressure, the maximum enlargement of the initial 30 nm grains up to 120 nm, with the sintering temperature increasing to 1800 °C, is consistent with the concept of slowing the growth when the submicron crystal size is reached [36].

The absence of graphite diffraction peaks in the diffraction patterns of all the synthesized samples convincingly demonstrates that the decomposition of dodecamethylhexasilinane under pressure proceeds with the predominant formation of hydrocarbons, and without molecular hydrogen and free carbon. Otherwise, we would observe the noticeable formation of graphite or diamond mixed with SiC.

### 3.2. Optical Properties

#### 3.2.1. Raman Spectra and IR Transmission

When studying optical properties, the main emphasis was placed on powders with an average particle size of ~100–300 nm, obtained at temperatures of 1350–2000 °C. This is due to the fact that the role of inhomogeneous broadening in optical spectra is of secondary importance for such powders, which allows a sequential (joint) analysis of IR transmission, Raman scattering, and luminescence spectra. In addition, at temperatures higher than 1300 °C, SiC particles do not show the tendency to agglomerate; therefore, they may be regarded as a more perspective material for IR photonics [31].

**Figure 3:**
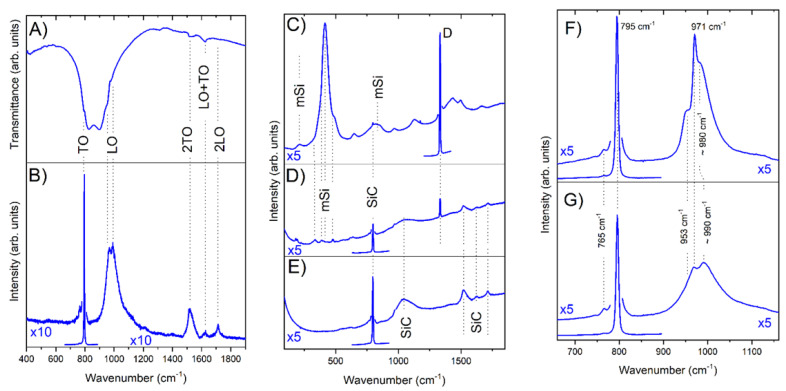
(**A**) IR transmission spectra of SiC crystals near lattice resonances. (**B**) Raman spectra upon excitation of the crystal by radiation with a wavelength of 632 nm. (**C**–**E**) Raman spectra for the following individual particles found in the sample synthesized in Ti capsule at 1600 °C: (**C**) a particle, mainly consisting of a diamond phase and metastable silicon; (**D**) a particle comprising SiC, a diamond phase, and metastable silicon; (**E**) silicon carbide crystal. Raman spectra for submicron particles, synthesized at 1350 °C, before (**F**) and after annealing (**G**) in the region corresponding to the first-order processes with participation of optical phonons. The spectra in Panel (**C**–**G**) were recorded using 532 nm excitation.

Figure 3A illustrates a typical IR transmission spectrum of SiC powder, recorded at room temperature, using a Bruker Optics IFS 66V IR Fourier-transform spectrometer (spectral resolution: 4 cm^−1^). To measure the transmission spectra, tablets containing 200 mg of pure KBr and 4 mg of SiC were prepared. The spectrum contains a dip characteristic of SiC, which corresponds to the region located between the LO and TO resonances. In the high-frequency region, absorption peaks associated with second-order processes are clearly recorded [37]. In addition, on the high-frequency part of the strong lattice reflection region, weak dips are observed in their spectral position, close to the frequency of the longitudinal optical (LO) phonons. Those dips can be attributed to the surface plasmon polaritons that are characteristic of doped (containing free carriers) 3C SiC particles [29].

The Raman spectra were measured using the following two devices: a portable EnSpectr R532 Raman spectrometer coupled to an Olympus S41 optical microscope, and a laboratory Raman spectrograph equipped with a cooled CCD detector. In the first case, a laser beam (wavelength λ = 532 nm), passing through an objective lens, was focused onto a sample placed on an adjustable stage. The Raman signal falling within the range of Stokes shifts (150–4000 cm^−1^) was recorded by a CCD matrix in the backscattering geometry with a 4 cm^-1^ spectral resolution. The typical excitation laser power was 1–5 mW, and the spot size, determined by the selected objective lens, was varied from 2 to 10 μm. As for the laboratory spectrograph, single-frequency lasers, operating at 472 nm, 532 nm, and 632 nm, were used as excitation sources. The excitation spot diameter on the sample was ~2 μm. The spectral resolution was 1 cm^−1^. The laboratory spectrograph also enabled measurements at low temperatures. Different wavelengths were required to separate the Raman and luminescence signals. The characteristic form of the Raman spectra of SiC powders did not practically depend on the selected excitation wavelength.

Figure 3B illustrates a typical Raman spectrum in synthesized micropowders. The most intense peaks are related to the first-order processes, involving transverse optical (TO) and longitudinal optical (LO) phonons corresponding to the Г point of the Brillouin zone [38]. The spectral position of the peak corresponding to the TO phonon (~795 cm^−1^) indicates that the crystals studied belong to the 3C polytype. This conclusion is consistent with the results of the X-ray structural analysis. In the region of the LO phonons, a fine structure is recorded, which is shifted to longer wavelengths with respect to the line characteristic of the LO (Г) phonons of 3C SiC. This behavior, which indicates the formation of mixed phonon–plasmon modes, independently confirms the presence of a noticeable concentration of free carriers in the synthesized crystals [39].

In the 1300–1800 cm^-1^ range, peaks at 1512 cm^−1^, 1626 cm^−1^, and 1712 cm^−1^ are recorded, which correspond to the second-order Raman processes [40]. The spectral position of these peaks is consistent with the results of the IR transmission spectra measurements in Figure 3A. The lowest frequency peak (2TO) arises due to the scattering of two transverse optical phonons related to the L point of the SiC Brillouin zone. The middle peak (LO + TO) is the least intense; it is not an overtone of the optical phonon modes, and it corresponds to a combination of phonons of different branches. The high-frequency peak (2LO) is the result of the scattering of two transverse optical phonons, with quasimomenta near the M point of the Brillouin zone [41].

Figure 3C–E illustrate a typical view of the Raman spectra of the micropowders obtained in the Ti capsule at 1600 °C. If XRD characterizes the sample bulk as a single SiC 3C phase (see Figure 2), micro-Raman investigations allow us to examine local variations in the sample composition. The Raman spectrum in Figure 3C shows the formation of diamond (D) [42] and metastable silicon (mSi) as impurity phases [43]. Figure 3D illustrates the presence of diamond and various phases of amorphous silicon, along with silicon carbide in the analyzing spot. Finally, Figure 3E illustrates the spectrum of silicon carbide microcrystals, free of the diamond (graphite) phase and metastable silicon phases. In general, the data presented in Figure 2 allow possible by-products of the synthesis of silicon carbide to be traced from dodecamethylhexasilinane.

To study the structural features of the synthesized SiC, a series of experiments were carried out to establish the effect of annealing in a vacuum on the Raman spectra. Examples of Raman spectra, before and after annealing in the region corresponding to the first-order scattering by optical phonons, are illustrated in Figure 3F,G. As can be seen from the figures, the spectrum in the TO phonon region does not change after annealing, and exhibits an intense peak in the region of 795 cm^−1^, which is typical for the 3C polytype. Nevertheless, in the 765 cm^−1^ region, a weak structure is recorded, the intensity of which is ~300–500 times less than the main (TO) peak intensity. Before annealing, a doublet structure is observed in the region of scattering by the LO phonons. The high-frequency (~990 cm^−1^) component of this structure corresponds to the mixed phonon–plasmon modes in doped 3C SiC, and the low-frequency component is located a few inverse centimeters below the LO (Г) resonance in the undoped 3C SiC. This fine structure is not associated with (possible) luminescent centers, and is retained in the Raman spectra of 100 nm individual crystals (see Figure S4 of Supplementary Materials). Similar behavior of the Raman spectra in the LO phonons region is observed in the case of nano- or submicron particles of 3C silicon carbide, in which a heavily doped region (core) is surrounded by a SiC layer (shell) depleted in carriers [29]. In this case, the formation of phonon–plasmon coupled modes, in combination with size effects, can explain the low-frequency and high-frequency components near LO resonance [29].

It should be noted that in the crystals synthesized at 1100 °C, there is no fine structure in the Raman spectrum near LO phonon resonance (a relatively narrow line, with a maximum at 970 cm^−1^, is observed). This means that no regions with a high carrier concentration are formed in ~5 nm particles. Therefore, one can argue that the region with a high carrier concentration (core) is formed in the particle center if its radius exceeds the depth of the carrier-depleted layer near the surface (shell). This situation can apparently be observed for relatively large particles synthesized at 1350 °C and above.

After annealing, the fine structure described above transforms into an asymmetric line with a maximum at 971 cm^−1^, which coincides with the frequencies of the LO (Г) phonons in undoped 3C SiC [38]. Thus, the tuning of the high-frequency LO peak component in Figure 3F corresponds to the transition from a mixed phonon–plasmon mode in the 3C-doped SiC, to scattering by conventional LO (Г) phonons in the pure 3C SiC [39]. Nevertheless, the long-wavelength structure observed in the spectra of the annealed crystals in the ~980 cm^−1^ region indicates the retention of regions in which the carrier concentration remains sufficiently high. The spectral position of the mixed phonon–plasmon mode enables the free carrier concentration to be estimated using the results of [29] (see Figures S5–S7 of Supplementary Materials). For the core of the as-grown particles, this concentration is at the level of 2–3 × 10^18^ cm^−3^, and drops to ~10^18^ cm^−3^ after annealing. It should be noted that for submicron-sized crystals, the mixed phonon–plasmon mode position depends on both the carrier concentration and the shape/internal structure/environment of the crystal. Therefore, the values obtained should be interpreted as estimates.

The presence of less intense, low-frequency components of the TO and LO phonon peaks could also be attributed to the presence of the 6H polytype in microcrystals. Nevertheless, with such an interpretation, one should expect comparable (and not differing by an order of magnitude) intensities for the TO and LO components, in the region of ~765 cm^−1^ and ~955 cm^−1^, respectively [38]. In addition, in the presence of other polytypes besides 3C, the Raman spectra should contain peaks in the region of 140–270 cm^−1^, which correspond to scattering by acoustic phonons [39]. In the studied crystals, those peaks were not observed. It should be noted that low-frequency shifts of the TO component can appear in the presence of stacking faults [44].

#### 3.2.2. Low-Temperature Photoluminescence

**Figure 4:**
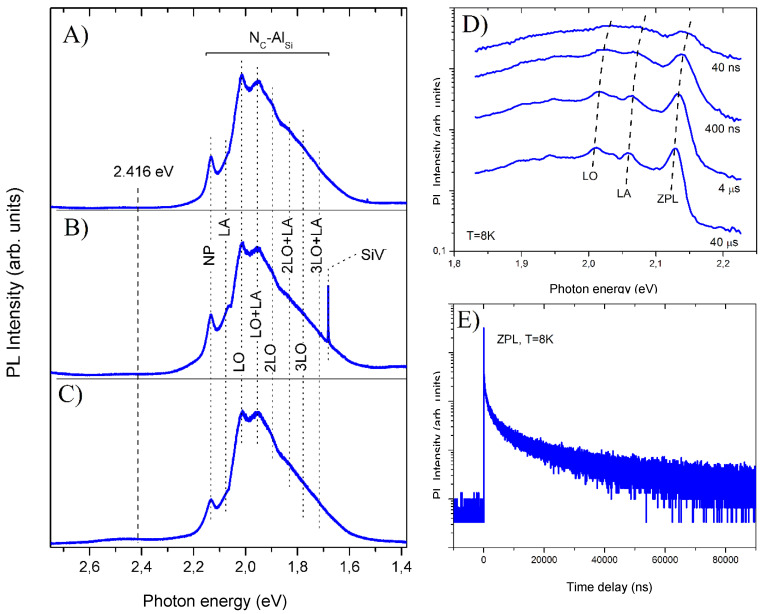
Examples of low-temperature (5K) photoluminescence spectra recorded for the as-grown (panels **A**,**B**) and annealed crystals (panel **C**). Panel (**A**) corresponds to the synthesis conditions in which the formation of side phases was not detected. Panel (**B**) corresponds to the situation in which metastable silicon and diamond were formed along with SiC during the synthesis. The dotted line marks the position of the fundamental absorption edge of 3C SiC. (Panels **D**,**E**) show time-resolved PL spectra and PL kinetics corresponding to the steady-state spectrum in Panel (**A**).

To assess the impurity-defect composition and determine the main reason for the appearance of free carriers, a series of experiments were carried out to measure the low-temperature photoluminescence spectra of the synthesized crystals. Figure 4 illustrates typical examples of such spectra, recorded for the original (panels A,B) and annealed crystals (panel C). Panel A in Figure 4 corresponds to the synthesis conditions under which the formation of side phases is not observed. Panel B corresponds to the situation in which metastable silicon and diamond are formed, along with SiC powders. In this case, the presence of diamond leads to the appearance of a narrow emission line of SiV centers in the PL spectra [45].

Except for the SiV emission line in all the spectra in Figure 4A–C, a similar structure is observed. This structure consists of a zero-phonon line (ZPL) near 2.13 eV, and its phonon replicas, with the participation of LO and LA phonons, with a quasimomentum located at the edge of the SiC Brillouin zone [46]. This structure was unambiguously assigned to the emission of the donor–acceptor pairs, with the nitrogen on the carbon sublattice (N_C_) and the aluminum on the silicon sublattice (Al_Si_) in the 3C polytype [47]. In our experiments, the assignment of this line to the donor–acceptor pairs was confirmed by measurements of the luminescence kinetics, as well as time-resolved luminescence spectra. In particular, ZPL reveals the long-wavelength shift of the emission lines with an increasing time delay and non-exponential intensity decay (see Figure 4D,E). This behavior, being a unique feature of donor–acceptor pairs, is governed by the spread of the distances between acceptors and donors [47].

On the one hand, the high intensity of the emission band of donor–acceptor pairs, in comparison with the excitation emission near the fundamental absorption edge of the 3C SiC (marked in Figure 4 with a dashed line), confirms the noticeable concentration of the corresponding point defects. On the other hand, the absence of any additional defect-related bands indicates that N_C_ and Al_Si_ are dominant types of shallow defects in the synthesized crystals. The nitrogen on the carbon sublattice in 3C SiC forms a donor center, the ground state energy of which is 53 MeV. At room temperature, this value is comparable to thermal energy; therefore, a significant part of the donor center turns out to be ionized. At the same time, the ground state energy for the Al_Si_ acceptor is 257 MeV, which is sufficient to significantly suppress their ionization at room temperature. Thus, it can be argued that the main reason for the appearance of free carriers in synthesized crystals is the ionization of the N_C_ donors. It should be noted that the electron concentrations of ~1–3 × 10^18^ cm^−3^, estimated above, exclude the reliable registration of donor impurities by the EDX method.

## 4. Conclusions

Thus, the HTHP synthesis of 3C silicon carbide powders from dodecamethylhexasilinane was carried out. It has been shown that by increasing the synthesis temperature from 800 °C to 1400 °C, it is possible to monotonically regulate the average crystallite size from ~2 nm to ~500 nm. At higher temperatures, further enlargement of the crystals is impeded, which is consistent with the recrystallization mechanism driven by a decrease in the surface energy of the particles. The main point defects in the synthesized crystals are aluminum impurities on the silicon sublattice Al_Si_ (acceptor) and nitrogen impurities on the carbon sublattice N_C_ (donor), but the concentrations of Al and N_C_ are outside the EDX sensitivity range. The Raman spectra, as well as the IR absorption spectra, indicate that the submicron particles obtained at 1350–2000 °C contain regions with both significant carrier concentration and depletion. Apparently, an increase of ~2–3∙10^18^ cm^−3^ in the concentration of carriers is characteristic of the core, surrounded by a depleted SiC layer (shell). We attribute the appearance of a noticeable concentration of free carriers to the relatively shallow N_C_ donor formation. When the synthesis conditions deviate from the optimum, the growth of 3C SiC competes with the formation of diamond and metastable silicon.

It is shown that the annealing of the SiC powders, obtained in a vacuum at ~1500 °C, leads to a noticeable (at least several times) decrease in the concentration of free carriers, while keeping Al_Si_ and N_C_ as the dominant point defects.

In general, the results obtained indicate that the developed synthesis technology, in combination with subsequent annealing in the vacuum, enables control over not only the size of the 3C SiC crystals, but also the properties of the localized phonon–plasmon resonance within the 3C SiC crystals. The latter feature may be of interest for the creation of hybrid systems with near-field resonant coupling in the mid-IR range.

## Figures and Tables

**Table 1:** Synthesis temperature (T), lattice parameter (Rietveld analysis), and crystallite size (Scherrer formula) for various SiC samples. Example of crystal size distribution at 2000 °C is given in Figure S2 of Supplementary Materials.

T, C	a, Å (Rietveld Method)	Size (Scherrer Formula), nm	Measured, nm
2000	4.357	200	100–500
1600	4.359	200	100–500
1400	4.359	150	100–500
1250	4.361	22	20–50
1100	4.367	5	<10
900	4.390	2	
800	4.397	1.6	

## Supplementary Materials

The following are available online at https://www.mdpi.com/article/10.3390/nano11113111/s1, Figure S1: X-ray characteristic spectrum of a sample obtained at 2000 °C; Figure S2: Statistical analysis of one of the characteristic samples synthesized at 2000 °C; Figure S3: The SEM photo of the sample synthesized at 2000 °C; Figure S4: Raman spectra of individual SiC particles obtained with different laser excitation wavelengths; Figure S5: Examples of calculated Raman spectra for “core-shell” particles depending on the plasma frequency; Figure S6: Calculated Raman spectra for core-shell particles depending on R_cs_; Figure S7: Calculated Raman spectra for core-shell particles as a function of γ_e_.

## References

[1] Anggara B.S., Fahdiran R., Marpaung M.A., Soegijono B. (2019). Silicon Carbide (SiC) Effect on Mechanical Properties and Corrosion Rates on Composite Al/SiC and Al-Cu/SiC.

[2] Davidsson J., Ivády V., Armiento R., Ohshima T., Son N.T., Gali A., Abrikosov I.A. (2020). Erratum: Identification of divacancy and silicon vacancy qubits in 6H-SiC. Appl. Phys. Lett..

[3] Zhu Y., Kovos B., Onizhuk M., Awschalom D., Gal G.L. (2021). Theoretical and experimental study of the nitrogen-vacancy center in 4H-SiC. Phys. Rev. Mater..

[4] Su J., Yang Y., Ren J., Guo P. (2020). Study on magnetic properties of Fe-doped 3C-SiC nanowires. J. Cryst. Growth.

[5] Yu Y., Liyan L., Guifu D., Ting C. (2019). SiC nanowire-based SU-8 with enhanced mechanical properties for MEMS structural layer design. Nanotechnol. Precis. Eng..

[6] Mi Y., Chen Y., Zheng Z., Qiao N., Liang Y. (2020). New discoveries in the growth of SiC whiskers derived from hydrogen silicone oil. J. Cryst. Growth.

[7] Károly Z., Mohai I., Klébert S., Keszler A., Sajó I.E., Szépvölgyi J. (2011). Synthesis of SiC powder by RF plasma technique. Powder Technol..

[8] Wu R., Zhou K., Yue C.Y., Wei J., Pan Y. (2015). Recent progress in synthesis, properties and potential applications of SiC nanomaterials. Prog. Mater. Sci..

[9] Dai D., Zhang N., Zhang W., Fan J. (2012). Highly bright tunable blue-violet photoluminescence in SiC nanocrystal-sodium dodecyl sulfonate crosslinked network. Nanoscale.

[10] Cooper O., Wang B., Brown C.L., Tiralongo J., Iacopi F. (2016). Toward Label-Free Biosensing with Silicon Carbide: A Review. IEEE Access.

[11] Wright N.G., Horsfall A.B. (2007). SiC sensors: A review. J. Phys. D Appl. Phys..

[12] Chaira D., Mishra B.K., Sangal S. (2007). Synthesis and characterization of silicon carbide by reaction milling in a dual-drive planetary mill. Mater. Sci. Eng. A.

[13] Real C., Alcala D., Criado J.M. (1997). Synthesis of silicon carbide whiskers from carbothermal reduction of silica gel by means of the constant rate thermal analysis (CRTA) method. Solid State Ion..

[14] Pan S., Zhang J., Yang Y., Song G. (2008). Effect of process parameters on the production of nanocrystalline silicon carbide from water glass. Ceram. Int..

[15] Narciso-Romero F.J., Rodrıguez-Reinoso F., Dıez M.A. (1999). Influence of the carbon material on the synthesis of silicon carbide. Carbon.

[16] Yang Y., Lin Z.M., Li J.T. (2009). Synthesis of SiC by silicon and carbon combustion in air. J. Eur. Ceram. Soc..

[17] Ebadzadeh T., Marzban-Rad E. (2009). Microwave hybrid synthesis of silicon carbide nanopowders. Mater. Charact..

[18] Narisawa M., Shimoda M., Okamura K., Sugimoto M. (1995). Reaction Mechanism of the Pyrolysis of Polycarbosilane and Polysilazane as Ceramic Precursors. Bull. Chem. Soc. Jpn..

[19] Čerović L., Milonjić S.K., Zec S.P. (1995). A comparison of sol-gel derived silicon carbide powders from saccharose and activated carbon. Ceram. Int..

[20] Cao L.Z., Jiang H., Song H., Liu X., Guo W.G., Yu S.Z., Li Z.M., Miao G.Q. (2010). SiC/SiO_2_ core–shell nanowires with different shapes: Synthesis and field emission properties. Solid State Commun..

[21] Schaaf P., Kahle M., Carpene E. (2005). Reactive laser synthesis of carbides and nitrides. Appl. Surf. Sci..

[22] Rai P., Park J.S., Park G.G., Lee W.M., Yu Y.T., Kang S.K., Moon S.Y., Hong B.G. (2014). Influence of carbon precursors on thermal plasma assisted synthesis of SiC nanoparticles. Adv. Powder Technol..

[23] Sasaki Y., Nishina Y., Sato M., Okamura K. (1987). Raman study of SiC fibres made from polycarbosilane. J. Mater. Sci..

[24] Ko S.M., Koo S.M., Cho W.S., Hwnag K.T., Kim J.H. (2012). Synthesis of SiC nano-powder from organic precursors using RF inductively coupled thermal plasma. Ceram. Int..

[25] Voronin G.A., Pantea C., Zerda T.W., Ejsmont K. (2001). Oriented growth of β-SiC on diamond crystals at high pressure. J. Appl. Phys..

[26] Ekimov E.A., Sadykov R.A., Gierlotka S. (2004). A High-Pressure Cell for High-Temperature Experiments in a Toroid-Type Chamber. Instrum. Exp. Tech..

[27] Zhong Y., Malagari S.D., Hamilton T., Wasserman D., Malagari D. (2015). Review of Mid-Infrared Plasmonic Materials. http://nanophotonics.spiedigitallibrary.org/.

[28] Bohren C.F., Wickramasinghe N.C. (1977). On the Computation of Optical Properties of Heterogeneous Grains. Astrophys. Space Sci..

[29] Sasaki Y., Nishina Y., Sato M., Qkamura K. (1989). Optical-phonon states of SiC small particles studied by Raman scattering and infrared absorption. Phys. Rev. B.

[30] Feng K., Streyer W., Islam S.M., Verma J., Jena D., Wasserman D., Hoffman A.J. (2015). Localized surface phonon polariton resonances in polar gallium nitride. Appl. Phys. Lett..

[31] Krivobok V.S., Kondorskiy A.D., Pashkeev D.A., Ekimov E.A., Shabrin A.D., Litvinov D.A., Grigoreva L.N., Kolosov S.A., Chernopitsskii M.A., Klekovkin A.V. (2021). A Hybrid Mid-IR Photodetector Based on Semiconductor Quantum Wells. Tech. Phys. Lett..

[32] Yang B., Wu T., Yang Y., Zhang X. (2017). Effects of charges on the localized surface phonon polaritons in dielectric nanoparticles. J. Opt. Soc. Am. B.

[33] Kondrina K.M., Kudryavtsev O.S., Vlasov I.I., Khmelnitskiy R.A., Ekimov E.A. (2018). High-pressure synthesis of microdiamonds from polyethylene terephthalate. Diam. Relat. Mater..

[34] Leite E.R., Ribeiro C. (2012). Crystallization and Growth of Colloidal Nanocrystals.

[35] Ekimov E.A., Kondrin M.V., Krivobok V.S., Khomich A.A., Vlasov I.I., Khmelnitskiy R.A., Iwasaki T., Hatano M. (2019). Effect of Si, Ge and Sn dopant elements on structure and photoluminescence of nano- and microdiamonds synthesized from organic compounds. Diam. Relat. Mater..

[36] Gubicza J., Nauyoks S., Balogh L., Labar J., Zerda T.W., Ungár T. (2007). Influence of sintering temperature and pressure on crystallite size and lattice defect structure in nanocrystalline SiC. J. Mater. Res..

[37] Hofmeister A.M., Pitman K.M., Goncharov A.F., Speck A.K. (2009). Optical constants of silicon carbide for astrophysical applications. II. Extending optical functions from infrared to ultraviolet using single-crystal absorption spectra. Astrophys. J..

[38] Aksyanov I.G., Kompan M.E., Kul’kova I.V. (2010). Raman scattering in mosaic silicon carbide films. Phys. Solid State.

[39] Yugami H., Nakashima S., Mitsuishi A., Uemoto A., Shigeta M., Furukawa K., Suzuki A., Nakajima S. (1987). Characterization of the free-carrier concentrations in doped β-SiC crystals by Raman scattering. J. Appl. Phys..

[40] Rohmfeld S., Hundhausen M., Ley L. (1998). Raman scattering in polycrystalline 3C-SiC: Influence of stacking faults. Physical Review B.

[41] Ekimov E.A., Lyapin S.G., Grigoriev Y.v., Zibrov I.P., Kondrina K.M. (2019). Size-controllable synthesis of ultrasmall diamonds from halogenated adamantanes at high static pressure. Carbon.

[42] Xu Z., He Z., Song Y., Fu X., Rommel M., Luo X., Hartmaier A., Zhang J., Fang F. (2018). Topic review: Application of raman spectroscopy characterization in micro/nano-machining. Micromachines.

[43] Ekimov E.A., Sherin P.S., Krivobok V.S., Lyapin S.G., Gavva V.A., Kondrin M.V. (2018). Photoluminescence excitation study of split-vacancy centers in diamond. Phys. Rev. B.

[44] Kuwabara H., Yamada S. (1975). Free-to-Bound Transition in beta-SiC Doped with Boron. Phys. Stat. Sol..

[45] Bishop S.G., Freitas J.A. (1990). Photoluminescence characterization of cubic SiC grown by chemical vapor deposition on Si substrates. J. Cryst. Growth.

[46] Borowicz P., Gutt T., Małachowski T., Łatek M. Structural investigation of silicon carbide with micro-raman spectroscopy. Proceedings of the 2009 MIXDES-16th International Conference Mixed Design of Integrated Circuits & Systems.

[47] Bai S., Ke Y., Shishkin Y., Shigiltchoff O., Devaty R.P., Choyke W.J., Strauch D., Stojetz B., Dorner B., Hobgood D. (2002). Four current examples of characterization of silicon carbide. Mater. Res. Soc. Symp.-Proc..

