# Supplementary material for: Structural and Optical Properties of Silicon Carbide Powders Synthesized from Organosilane Using High-Temperature High-Pressure Method
Source: Nanomaterials (Basel). 2021 Nov 18;11(11):3111. doi: 10.3390/nano11113111 (PMC8617940; doi:10.3390/nano11113111)
Supplement: Supplementary file 1 [file nanomaterials-11-03111-s001.zip › nanomaterials-1437325-supplementary.pdf]

nanomaterials

Supplementary Materials

Structural and Optical Properties of Silicon Carbide Powders Synthesized from Organosilane Using High-temperature High-pressure Method

Evgeny A. Ekimov [1], Vladimir S. Krivobok [2,*], Mikhail V. Kondrin [1], Dmitry A. Litvinov [2], Ludmila N. Grigoreva [2,3], Aleksandra V. Koroleva [3], Darya A. Zazymkina [2], Roman A. Khmelnitskii [2], Denis F. Aminev [2] and Sergey N. Nikolaev [2]

[1] Institute for High Pressure Physics of the Russian Academy of Sciences, Kaluzhskoe shosse, 14, Troitsk, Moscow 108840, Russia; ekimov@hppi.troitsk.ru (E.A.E.); mkondrin@hppi.troitsk.ru (M.V.K.)
[2] P.N. Lebedev Physical Institute of the Russian Academy of Sciences, Leninsky Prospect, 53, Moscow 119991, Russia; litvinovd@lebedev.ru (D.A.L.); zazymkina_darya@mail.ru (D.A.Z.); roma@lebedev.ru (R.A.K.); amdenis@yandex.ru (D.F.A.); nikolaev-s@yandex.ru (S.N.N.)
[3] Lomonosov Moscow State University, Leninskiye Gory 1, Moscow 119991, Russia; ln.grigorjeva@physics.msu.ru (L.N.G.); koroleva.phys@mail.ru (A.V.K.)
* Correspondence: kolob7040@gmail.com

Example of EDX data for SiC powders

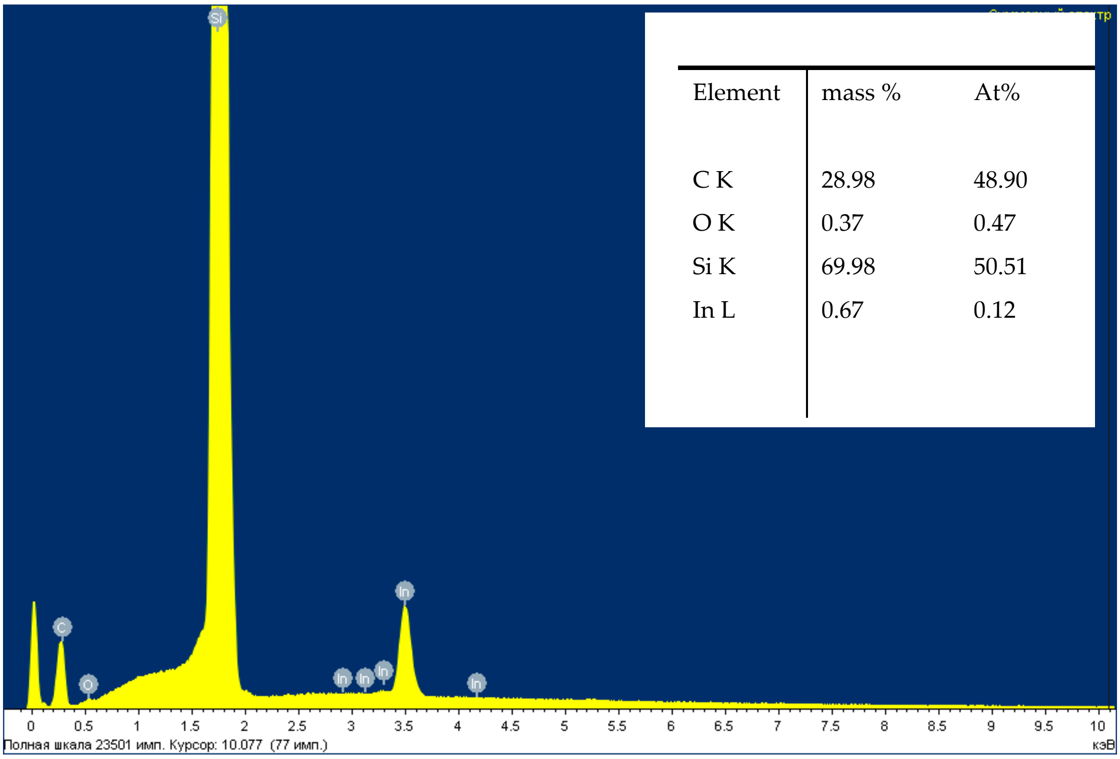

Element	mass %	At%
C K	28.98	48.90
O K	0.37	0.47
Si K	69.98	50.51
In L	0.67	0.12

Figure S1. X-ray characteristic spectrum of a sample obtained at 2000 °C. The sample was placed on the Indium plate, which is the reason for In detection in the spectrum.

Size distribution of SiC particles

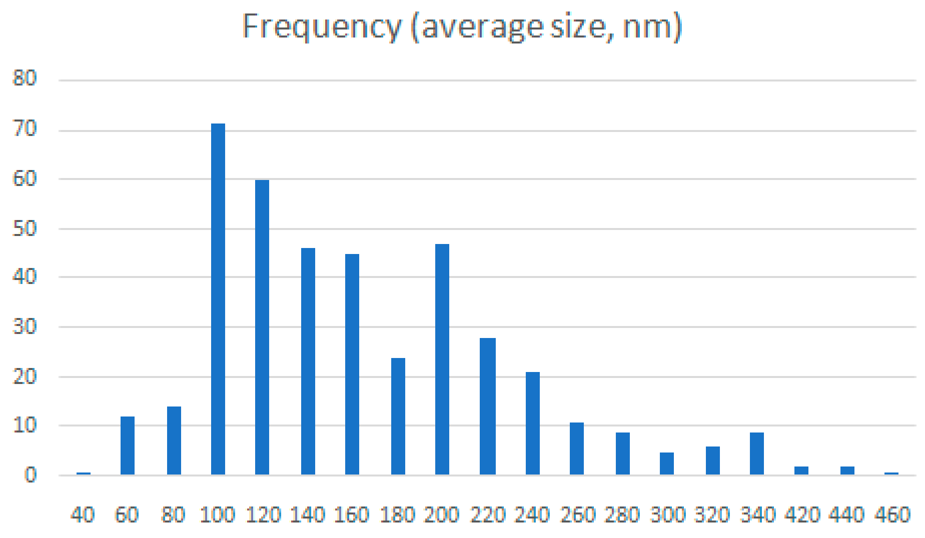

Figure S2. Statistical analysis of one of the characteristic samples synthesized at 2000 °C. The original SEM photo is shown in Fig. S3. Similar distributions we observed for samples obtained at 1400-2000 °C. It can be seen that ~ 90% particles vary in size in the range from 100 to 300 nm.

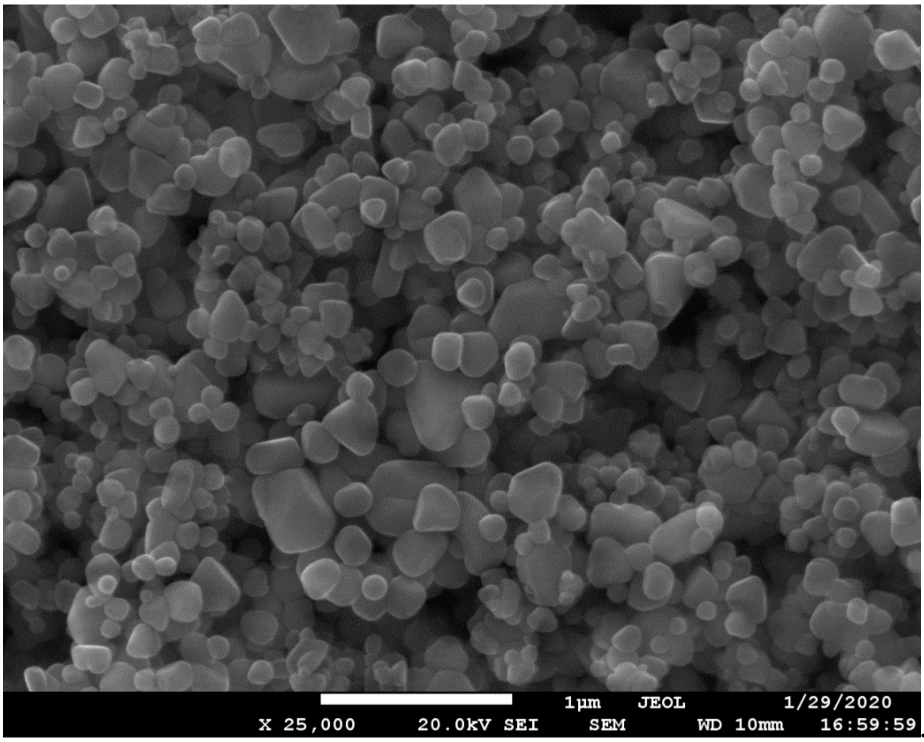

Figure S3. The SEM photo of the sample synthesized at 2000 °C.

Examples of Raman spectra for individual SiC particles

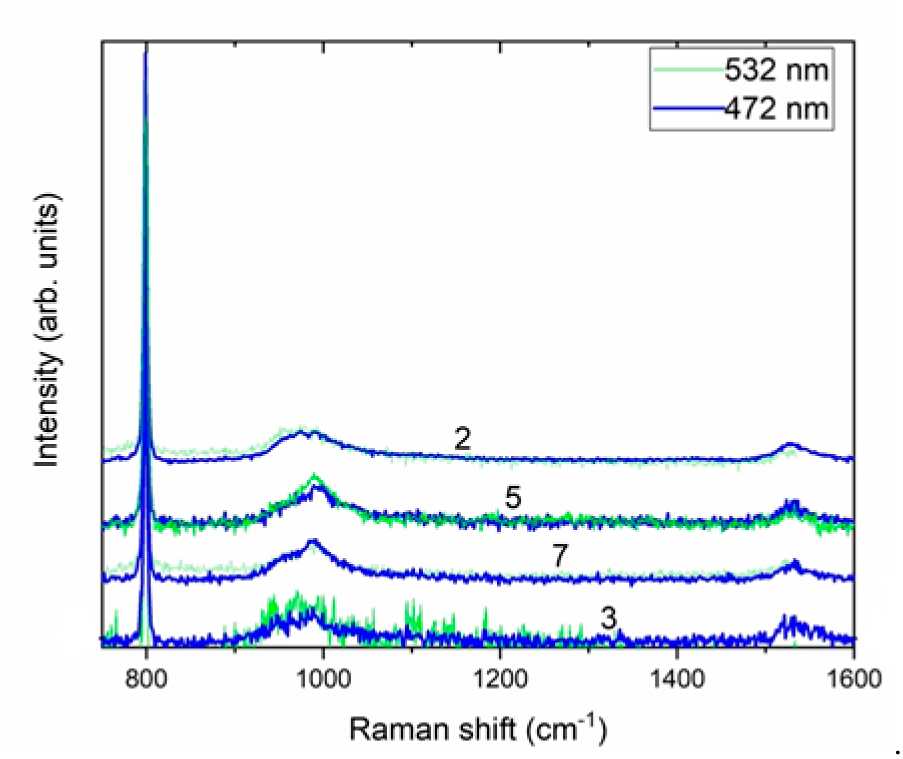

Figure S4. Raman spectra of individual SiC particles obtained with different laser excitation wavelengths (472 nm is marked with blue colour, 532 nm is the green one). The lowest one with number 3 presents the Raman spectra of the smallest ~100 nm nanoparticle. In each case complex structure near the LO resonance can be clearly seen. Temperature T=300K.

Modeling of Raman peaks related to coupled LO phonon-plasmon modes

In Figure. 3 of the manuscript, a fine structure is observed in the Raman spectra in the LO phonon region. This fine structure remains in the Raman spectra of individual particles, see e.g. Figure. S4. This means that there is an inhomogeneity in a single SiC particle. At the same time, the particles consist of the 3C SiC only as it follows from the Raman spectra (e.g. in the region of TO phonon peak) and from the X-ray data. So inhomogeneity is associated not with a change in the structure, but with the presence of regions characterized by different carrier concentrations. It is possible due to the formation of depleted layers near the surface, see e.g. Ref. [29] of manuscript. In this case, a model of a core plasmon and a carrier-free layer on the surface (shell) of the particles can be used to describe the Raman spectra in the region of mixed phonon-plasmon resonance, see eq. (15) in ref. [29]. In this model, the Raman spectrum near LO phonon peak depends on a number of parameters:

- the carrier concentration in the particle core, which determines the plasma frequency (w_P)
- damping associated with free carriers in the particle core (γ_e)
- longitudinal and transverse phonons frequencies (w_l and w_t)
- the ratio between the characteristic size of the core and shell (R_{cs}) of the particle
- the characteristic phonon damping constants in the core and shell (Γ_c and Γ_s), which are governed by anharmonic decay and electron-phonon interaction. These constants depend on the particle size and the carrier concentration in them.

In addition, the spectrum shape depends on the (random) dielectric environment of the particles.

Among the listed parameters, only w_l and w_t should be considered as universal constants, while all other parameters can vary for different particles, including those synthesized in the same experiment. Thus, it is impossible to accurately determine the above

parameters based on the experimental Raman spectra near LO phonon-plasmon resonances.

Figures S5,S7 show examples of the calculated Raman spectra for "core-shell" particles depending on the plasma frequency (S5), the R_{cs} ratio (S6), and the constant characterizing the damping in the electronic subsystem γ_e (S7). As can be seen from the figures, the position of the high-frequency peak associated with the mixed phonon-plasmon mode sharply depends on the parameter w_p and can only slightly shift to the low-frequency region when decreasing R_{cs}. In turn, γ_e slightly affects the high-frequency peak position, significantly affecting only its width. Also the position of the mentioned peak weakly depends on the parameters Γ_c and Γ_s when they vary in the range from several cm^{-1} to several tens cm^{-1} (characteristic values of broadening for optical phonons associated with their finite lifetimes). Consequently, it is possible to estimate the range of w_p values via high-frequency peak spectral position. Since the high-frequency peak of the as-grown particles is located in the 980–990 cm^{-1}, it can be argued that w_p can vary in the range of 500–900 cm^{-1}. This corresponds to the ~ 2–3 × 10^{18} cm^{-3} carrier concentration in the particle core.

For the shell carrier concentration only a rough estimate is possible. According to our data, to register a well-resolved low-frequency peak in the region of 940–950 cm^{-1}, it is necessary that the shell carrier concentration be several units per 10^{17} cm^{-3} or lower.

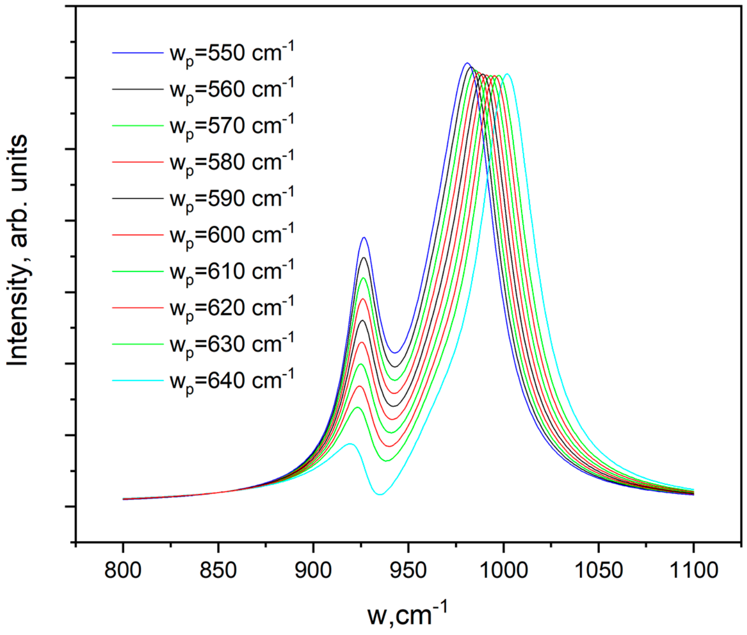

Figure S5. Examples of calculated Raman spectra for "core-shell" particles depending on the plasma frequency for the following values of the parameters γ_e =100cm^{-1} , w_l =969 cm^{-1}, w_t =793cm^{-1}, R_{cs} =0.95, Γ_c =20 cm^{-1}, Γ_s =50 cm^{-1}.

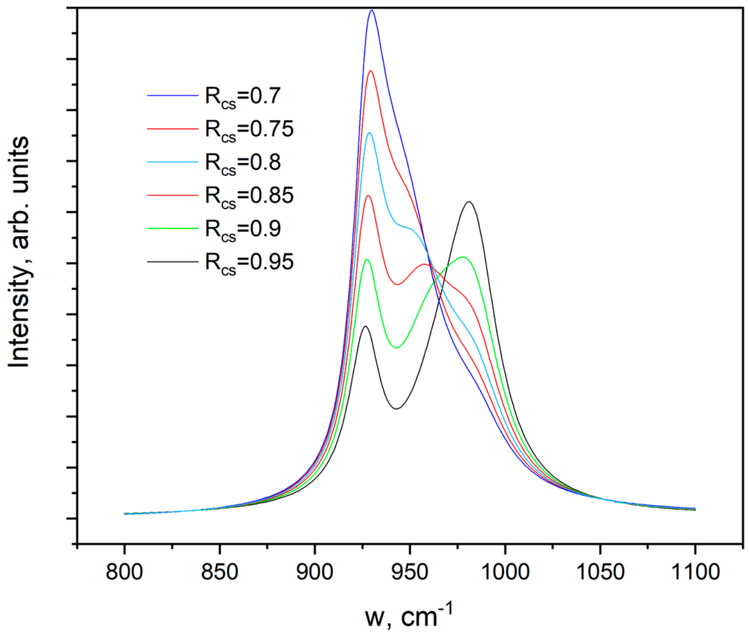

Figure S6. Calculated Raman spectra for core-shell particles depending on R_{cs} for the following values of other parameters γ_e = 100 cm^{-1}, w_l =969 cm^{-1}, w_t =793 cm^{-1}, w_p =550 cm^{-1}, Γ_c =20 cm^{-1}, Γ_s =50 cm^{-1}.

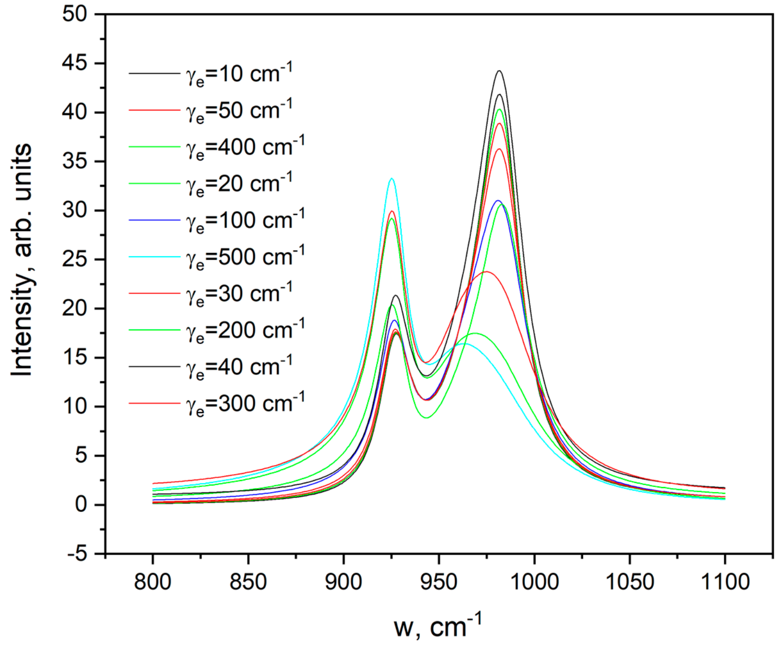

Figure S7. Calculated Raman spectra for core-shell particles as a function of γ_e for the following values of the parameters R_{cs} =0.95 , w_l =969 cm^{-1}, w_t =793 cm^{-1}, w_p =550 cm^{-1}, Γ_c =20 cm^{-1}, Γ_s =50 cm^{-1}.
